# Emergency Department Process Times and Door-In–Door-Out Times in Interhospital Transfers After Acute Ischemic Stroke

**DOI:** 10.1001/jamanetworkopen.2024.31183

**Published:** 2024-09-03

**Authors:** Regina Royan, Brian Stamm, Mihai Giurcanu, Steven R. Messe, Edward C. Jauch, Shyam Prabhakaran

**Affiliations:** 1 Department of Emergency Medicine, University of Michigan, Ann Arbor; 2 Institute for Healthcare Policy and Innovation, University of Michigan, Ann Arbor; 3 Department of Neurology, University of Michigan, Ann Arbor; 4 Lieutenant Colonel Charles S. Kettles Veterans Affairs Medical Center, Ann Arbor, Michigan; 5 Department of Public Health Sciences, University of Chicago, Chicago, Illinois; 6 Department of Neurology, University of Pennsylvania, Philadelphia; 7 Department of Research, Mountain Area Health Education Center, Asheville, North Carolina; 8 Department of Neurology, University of Chicago, Chicago, Illinois

## Abstract

**Importance:**

Stroke treatment is exquisitely time sensitive. The door-in–door-out (DIDO) time, defined as the total time spent in the emergency department (ED) at a transferring hospital, is an important quality metric for the care of acute stroke. However, little is known about the contributions of specific process steps to delays and disparities in DIDO time.

**Objective:**

To quantify process steps and their association with DIDO times at transferring hospitals among patients with acute ischemic stroke (AIS).

**Design, Setting, and Participants:**

This retrospective cohort study analyzed patients in the American Heart Association Get With the Guidelines–Stroke registry with AIS presenting between January 1, 2019, to December 31, 2021, and transferred from the presenting hospital ED to another acute care hospital for evaluation of thrombolytics, endovascular therapy, or postthrombolytic care. Data were analyzed from July 8 to October 13, 2023.

**Exposures:**

Intervals of ED care of ischemic stroke: door-to-imaging and imaging-to-door times.

**Main Outcomes and Measures:**

The primary outcome was DIDO time. Multivariate generalized estimating equations regression models were performed to compare contributions of interval process times to explain variation in DIDO time, controlling for patient- and hospital-level characteristics.

**Results:**

Among 28 887 patients (50.5% male; mean [SD] age, 68.3 [14.8] years; 5.5% Hispanic, 14.7% non-Hispanic Black, and 73.2% non-Hispanic White), mean (SD) DIDO time was 171.4 (149.5) minutes, mean (SD) door-to-imaging time was 18.3 (34.1) minutes, and mean (SD) imaging-to-door time was 153.1 (141.5) minutes. In the model adjusting for door-to-imaging time, the following were associated with longer DIDO time: age 80 years or older (compared with 18-59 years; 5.97 [95% CI, 1.02-10.92] minutes), female sex (5.21 [95% CI, 1.55-8.87] minutes), and non-Hispanic Black race (compared with non-Hispanic White 10.09 [95% CI, 4.21-15.96] minutes). In the model including imaging-to-door time as a covariate, disparities in DIDO by age and female sex became nonsignificant, and the disparity by Black race was attenuated (2.32 [95% CI, 1.09-3.56] minutes).

**Conclusions and Relevance:**

In this national cohort study of interhospital transfer of patients with AIS, delays in DIDO time by Black race, older age (≥80 years), and female sex were largely explained by the imaging-to-door period, suggesting that future systems interventions should target this interval to reduce these disparities. While existing guidelines and care resources heavily focus on reducing door-to-imaging times, further attention is warranted to reduce imaging-to-door times in the management of patients with AIS who require interhospital transfer.

## Key Points

**Question:**

What are the key process steps in the care of patients with acute ischemic stroke (AIS) that contribute to the duration of time spent at transferring hospitals?

**Findings:**

This retrospective cohort study of a national, hospital-based registry included 28 887 patients with AIS. Imaging-to-door time contributed more to overall door-in–door-out time than other components of emergency care at transferring hospitals.

**Meaning:**

To improve the timeliness of interhospital transfer of patients with AIS, quality improvement efforts and care guidelines should focus on process steps that occur after imaging.

## Teaser

This cohort study quantifies the length of time to complete process steps in the initial care of patients with acute ischemic stroke and determines which process steps account for most disparities by sex, race and ethnicity, and age in door-in–door-out times for interhospital transfer.

## Introduction

Timely treatment can reduce disability due to acute ischemic stroke (AIS),^1,2^ and expeditious interhospital transfer is often required when advanced treatments, such as thrombolysis and endovascular therapy (EVT), are not offered at the hospital where a patient initially presents.^3^ The time from arrival to discharge from the emergency department (ED) at the transferring hospital is defined as the door-in–door-out (DIDO) time, an important quality metric.^4^ DIDO times for patients with large-vessel occlusions who may require EVT are often prolonged.^5,6,7^ A recent Get With The Guidelines (GWTG)–Stroke registry study found that DIDO times for patients with AIS transferred for EVT consideration from GWTG hospitals^7^ exceeded the recommendations of less than 120 minutes.^8^ Additionally, there were significant disparities in DIDO times, with female sex, Black race, and older age associated with significantly longer DIDO times.^7^ Given the time sensitivity of the cerebral ischemic penumbra, these significant delays in the transfer process can lead to worse clinical outcomes.^3,5,9,10,11^ The extent to which individual process steps affect DIDO time delays and disparities has not, to our knowledge, been fully evaluated in prior studies.

Since 2013, guidelines for the emergency management of AIS have recommended the achievement of specific time goals for ED-based stroke process metrics.^12,13^ For instance, a goal door-to-imaging time of 20 minutes or less has been recommended by the American Heart Association and American Stroke Association (AHA-ASA)^13^; quality improvement (QI) initiatives have targeted such metrics, and some literature suggests that door-to-imaging times have improved over time.^14^ Less attention has been paid to the process intervals that occur after initial brain imaging is complete,^15^ such as imaging-to-door time (time from imaging to ED departure). Furthermore, it is unknown which process intervals contribute most to disparities in DIDO times. Defining which acute stroke process steps have the greatest impact on DIDO times is essential to identify barriers to timely transfer and can help guide future national QI initiatives to optimize the workflow of acute stroke transfers. This study sought to (1) quantify the length of time to complete process steps in the initial care of patients with AIS and (2) determine which process steps account for most sex, race and ethnicity, and age disparities in DIDO times for interhospital transfer.

## Methods

Data were obtained from the GWTG-Stroke registry, an ongoing, national database for voluntary QI maintained by the AHA-ASA that includes all patients diagnosed with AIS who received care at the participating hospital and is generally representative of the US Medicare population with AIS.^16^ Each participating hospital received either human research approval to enroll patients without individual consent under the common rule or a waiver of authorization and exemption from subsequent review by their institutional review board (IRB). Advarra, the IRB for the AHA determined that this study was exempt from IRB oversight. This study follows Strengthening the Reporting of Observational Studies in Epidemiology (STROBE) reporting guidelines for cohort studies.

### Study Population

Patients in the GWTG-Stroke registry with AIS, presenting between January 1, 2019, and December 31, 2021, were included in this study if they were not admitted at the participating hospital and were transferred from the ED to another acute care hospital for evaluation of intravenous thrombolytic care, consideration of EVT, or postthrombolytic care. These inclusion criteria identify the patients with the most time-sensitive care needs.^1,2^ Specifically, “drip and ship” transfers (patients having already received thrombolysis at the transferring facility) were included, given that these transfers have been increasing over time,^17^ and many transferring hospitals lack the infrastructure necessary for postthrombolytic care, necessitating emergent transfer, which has been recognized and recommended by the Joint Commission.^18^ Patients were excluded if they were transferred from a comprehensive stroke center, had negative DIDO times, or had values greater than 72 hours. All patient data were collected from the GWTG transferring hospitals.

### Interval Process Times

**Figure 1.**
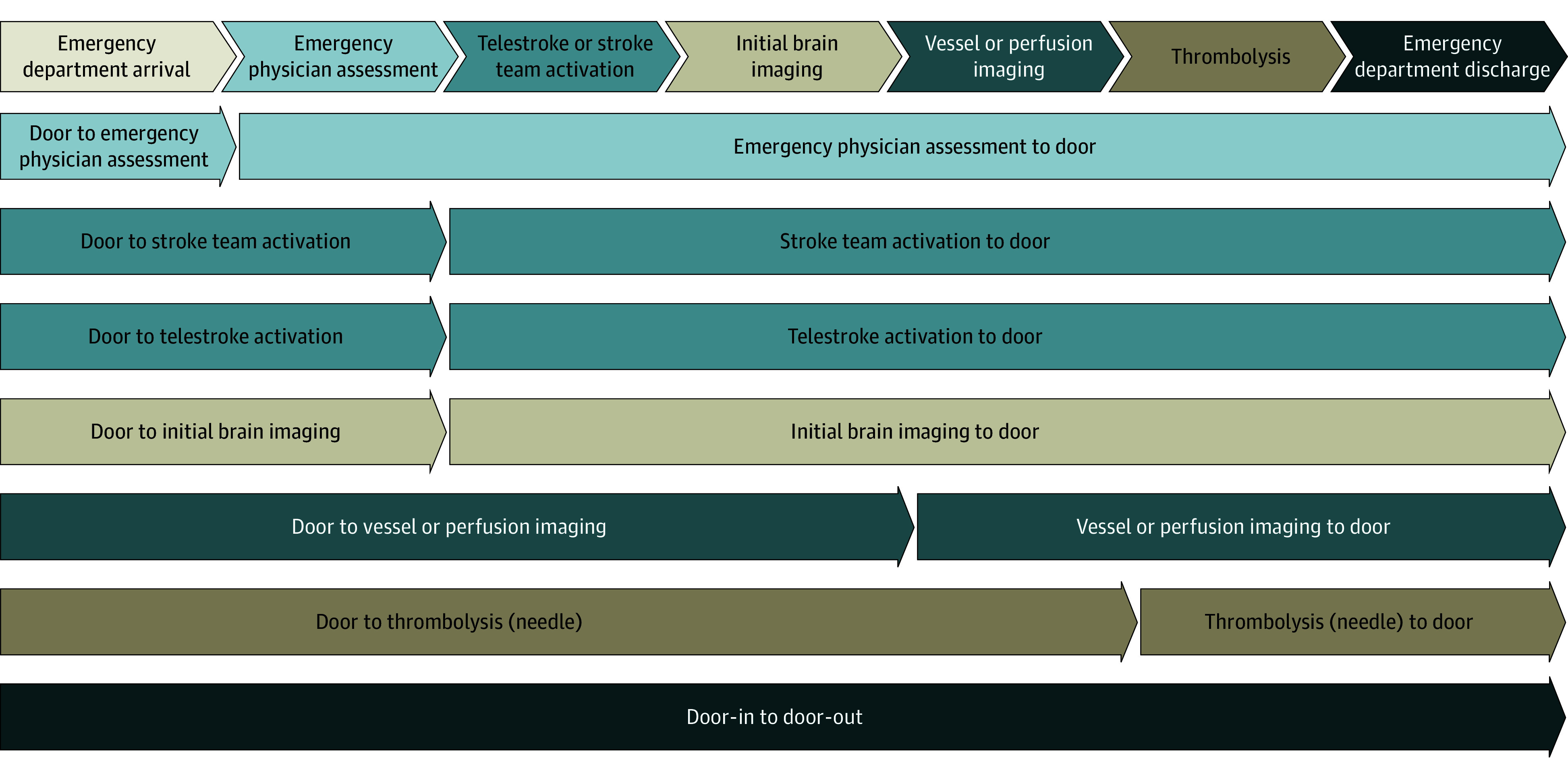
Component Intervals in Door-In–Door-Out Time

**Figure 2.**
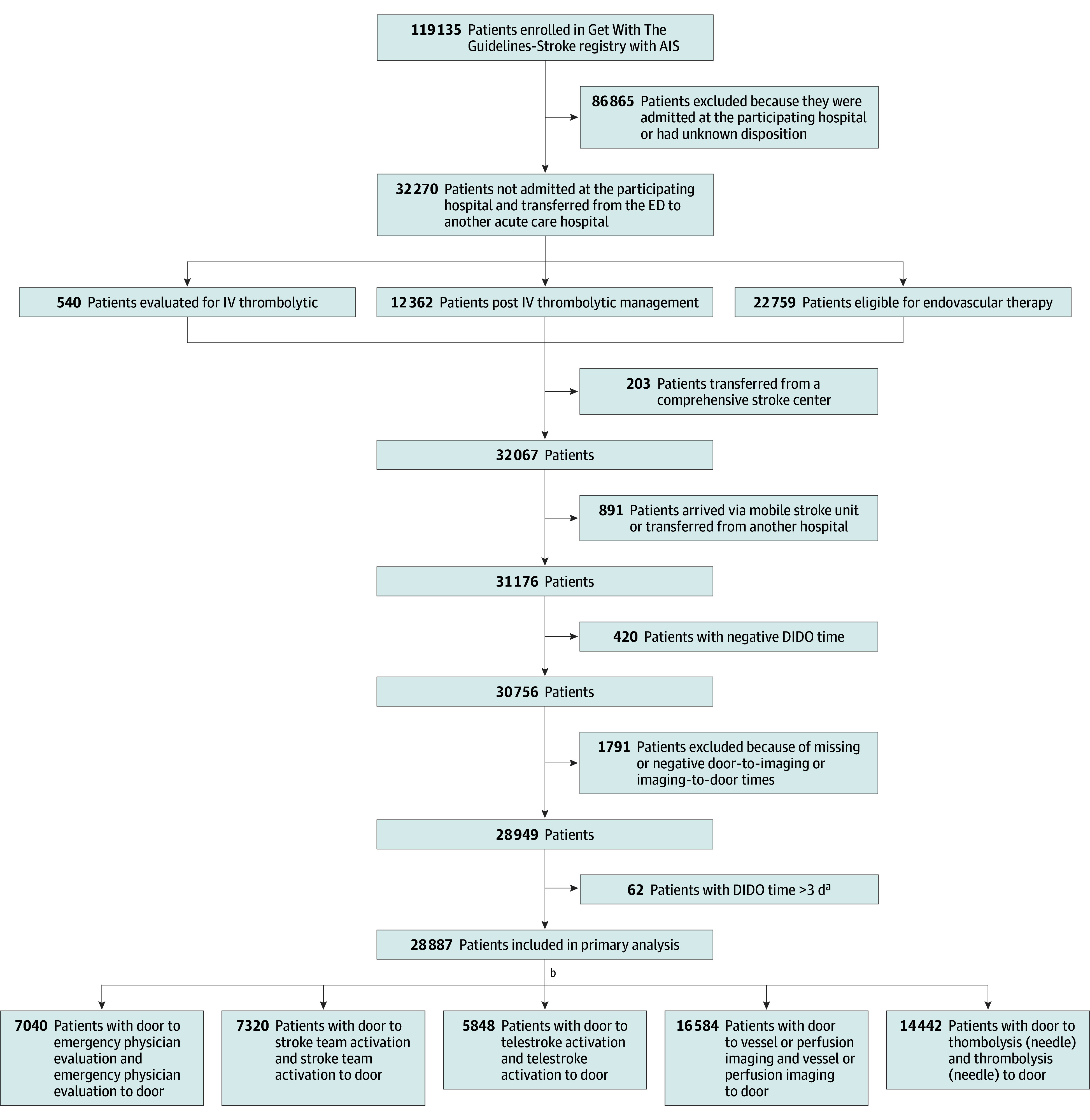
Study Population Flowchart AIS indicates acute ischemic stroke; DIDO, door-in–door-out; ED, emergency department; IV, intravenous. ^a^DIDO times greater than 3 days were excluded based on an assessment of the distribution of DIDO time and empirical interpretation that such outliers in DIDO time would likely represent atypical reasons for transfer such as family request, bed capacity constraints, or insurance (out of network) factors rather than clinical factors. There may be some, although relatively few, patients, with a DIDO time greater than 3 days who were excluded who had clinical reasons for transfer. ^b^Missingness varied for each of the following intervals included in eTables 2-6 in Supplement 1. Intervals were not mutually exclusive or sequential.

The primary exposures were door-to-imaging and imaging-to-door times. Secondary exposures were other interval process times, including door to emergency physician assessment, door to initiation of vessel or perfusion imaging, door to activation of the use of interactive video-conferencing (telestroke), door to activation of stroke team, and door to thrombolytic administration (door to needle). The corresponding subintervals from time of the respective process step to door-out (ED transfer) time were additionally calculated. The associations between these intervals and DIDO time are displayed in Figure 1. Figure 2 shows the flowchart describing the derivation of the cohort.

### Outcomes and Patient and Hospital Characteristics

The primary outcome was DIDO time (time of transfer out minus the time of arrival at the transferring hospital) measured as a continuous variable. Covariates included a priori specified patient, clinical, and hospital characteristics. Information was collected on indications for interhospital transfer (EVT, evaluation of thrombolytics, or postthrombolytic care).

Patient-level characteristics included age, sex, race and ethnicity, health insurance status, vascular risk factors and pertinent medical history (hypertension, diabetes, dyslipidemia, atrial fibrillation, prior stroke, prior transient ischemic attack, prosthetic heart valve, coronary artery disease or myocardial infarction, carotid artery stenosis, peripheral vascular disease, heart failure, and smoking), and prestroke antithrombotic medications. Data on race and ethnicity and insurance status were extracted from the medical record for inclusion in the registry. Categories of race and ethnicity included Hispanic, non-Hispanic Black, White non-Hispanic, and other (including American Indian or Alaska Native, Asian, Native Hawaiian or Other Pacific Islander, or unable to determine); race and ethnicity were included as covariates due to previously described disparities in DIDO times by race and ethnicity.^7^ Clinical and arrival characteristics included National Institutes of Health Stroke Scale (NIHSS) score, mode of arrival to ED such as emergency medical services (EMS) to the transferring hospital or private transport, use of EMS prenotification, arrival after hours defined as any time between 6 pm and 7 am on Monday through Friday or during the weekend (Saturday or Sunday),^19^ and whether the patient arrived during the COVID-19 pandemic (March 11, 2020, through December 31, 2021).^20^ Transferring hospital characteristics included stroke center certification status (primary stroke center vs acute stroke–ready and noncertified hospitals), hospital geographic location (rural vs urban), annual volume of thrombolysis for ischemic stroke, teaching status, and daily hospital census.

### Missing Data

All covariates had missingness of less than 10%, with the exception of insurance status (25.1%), as seen in Table 1. Complete case analysis was used for the analytic models, and insurance status was excluded due to high missingness.

**Table 1.** Characteristics of Patients With AIS Transferred for Evaluation and/or Management of Thrombolysis or Endovascular Therapy

Characteristic	Evaluation or management group, No. (%)					
	Received CT imaging (n = 28 887)	ED physician assessment (n = 7941)	Stroke team activation (n = 8422)	Telestroke activation (n = 7006)^a^	Received thrombolysis (n = 16 810)	Vessel or perfusion imaging (n = 19 151)
**Demographics**						
Age, y						
18-59	7736 (26.8)	2178 (27.4)	2250 (26.7)	1885 (26.9)	4864 (28.9)	5044 (26.3)
60-69	6724 (23.3)	1878 (23.6)	2014 (23.9)	1566 (22.4)	3886 (23.1)	4470 (23.3)
70-79	7193 (24.9)	1970 (24.8)	2119 (25.2)	1789 (25.5)	4153 (24.7)	4797 (25.0)
80-110	7234 (25.0)	1915 (24.1)	2039 (24.2)	1766 (25.2)	3907 (23.2)	4840 (25.3)
Sex						
Female	14 288 (49.5)	3932 (49.5)	4172 (49.6)	3394 (48.5)	8301 (49.4)	9379 (49.0)
Male	14 587 (50.5)	4005 (50.5)	4245 (50.4)	3611 (51.5)	8499 (50.6)	9764 (51.0)
Missing	12	4	5	1	10	8
Race and ethnicity						
Hispanic	1575 (5.5)	384 (4.8)	450 (5.3)	331 (4.7)	867 (5.2)	1097 (5.7)
Non-Hispanic Black or African American	4228 (14.7)	1050 (13.2)	1100 (13.1)	999 (14.3)	2303 (13.7)	2871 (15.0)
Non-Hispanic White	21 129 (73.2)	6075 (76.5)	6400 (76.0)	5268 (75.2)	12 555 (74.8)	13 884 (72.6)
Non-Hispanic Other^b^	1922 (6.7)	429 (5.4)	468 (5.6)	407 (5.8)	1053 (6.3)	1282 (6.7)
Missing	33	3	4	1	32	17
Insurance^c^						
Medicaid	809 (3.7)	218 (3.7)	203 (3.2)	193 (3.6)	473 (3.8)	473 (3.3)
Medicare	17 466 (80.7)	4772 (80.9)	5073 (81.1)	4283 (80.8)	9868 (79.5)	11 619 (82.1)
Private, VA, Champus, or other	2547 (11.8)	668 (11.3)	740 (11.8)	603 (11.4)	1603 (12.9)	1586 (11.2)
Self-pay or no insurance	635 (2.9)	189 (3.2)	193 (3.1)	185 (3.5)	384 (3.1)	376 (2.7)
Not determined	183 (0.8)	48 (0.8)	50 (0.8)	38 (0.7)	90 (0.7)	104 (0.7)
Missing	7247	2046	2163	1704	4392	4993
**Medical history**						
Hypertension	18 822 (65.6)	5151 (65.1)	5425 (64.7)	4572 (65.3)	10 842 (64.9)	12 512 (65.7)
Dyslipidemia	10 887 (37.9)	3065 (38.7)	3271 (39.0)	2683 (38.3)	6377 (38.2)	7387 (38.8)
Diabetes	7476 (26.1)	2064 (26.1)	2151 (25.7)	1784 (25.5)	4223 (25.3)	4893 (25.7)
Prior stroke	5254 (18.3)	1478 (18.7)	1553 (18.5)	1311 (18.7)	2678 (16.0)	3428 (18.0)
CAD or MI	5513 (19.2)	1556 (19.7)	1659 (19.8)	1395 (19.9)	3227 (19.3)	3589 (18.9)
Smoking	4392 (15.3)	1240 (15.7)	1299 (15.5)	1055 (15.1)	2581 (15.5)	2871 (15.1)
Atrial fibrillation	5182 (18.1)	1417 (17.9)	1551 (18.5)	1229 (17.6)	2285 (13.7)	3582 (18.8)
Heart failure	2445 (8.5)	700 (8.8)	752 (9.0)	599 (8.6)	1231 (7.4)	1680 (8.8)
Prior TIA	1889 (6.6)	569 (7.2)	608 (7.3)	520 (7.4)	1146 (6.9)	1240 (6.5)
Peripheral vascular disease	657 (2.3)	190 (2.4)	212 (2.5)	165 (2.4)	332 (2.0)	466 (2.4)
Carotid artery stenosis	678 (2.4)	242 (3.1)	219 (2.6)	154 (2.2)	372 (2.2)	473 (2.5)
Prosthetic heart valve	304 (1.1)	88 (1.1)	96 (1.1)	84 (1.2)	145 (0.9)	216 (1.1)
Missing	198	24	38	4	108	119
**Arrival and clinical data**						
Arrival mode						
Private	6947 (24.3)	2214 (28.0)	2002 (23.9)	1691 (24.3)	4300 (25.9)	4280 (22.6)
EMS without prenotification	5135 (18.0)	1350 (17.1)	1309 (15.6)	930 (13.3)	2659 (16.0)	3464 (18.3)
EMS with prenotification	16 490 (57.7)	4336 (54.9)	5077 (60.5)	4352 (62.4)	9674 (58.2)	11 228 (59.2)
Missing	315	41	34	33	177	179
During pandemic	17 454 (60.4)	4913 (61.9)	5215 (61.9)	4416 (63.0)	9868 (58.7)	12 260 (64.0)
NIHSS score^d^						
0-6	9577 (33.9)	2872 (36.8)	2730 (32.9)	2492 (35.9)	5779 (34.9)	6102 (32.5)
7-12	6461 (22.9)	1686 (21.6)	1847 (22.2)	1552 (22.3)	3880 (23.4)	4261 (22.7)
13-19	6200 (22.0)	1617 (20.7)	1853 (22.3)	1463 (21.1)	3589 (21.6)	4200 (22.4)
≥20	6003 (21.3)	1630 (20.9)	1873 (22.6)	1438 (20.7)	3334 (20.1)	4205 (22.4)
Missing	646	136	119	61	228	383
NIHSS stroke scale score, median (IQR)	10 (5-18)	10 (4-18)	11 (5-19)	10 (4-18)	10 (5-18)	11 (5-19)
**Transferring hospital characteristics**						
Certification status						
PSC	13 707 (47.5)	3400 (42.8)	3656 (43.4)	2968 (42.4)	7288 (43.4)	9522 (49.7)
Non-PSC^e^	15 180 (52.5)	4541 (57.2)	4766 (56.6)	4038 (57.6)	9522 (56.6)	9629 (50.3)
Location						
Rural	7867 (27.6)	2333 (29.8)	2310 (27.8)	2522 (36.5)	5214 (31.5)	4252 (22.4)
Urban	20 649 (72.4)	5496 (70.2)	6002 (72.2)	4393 (63.5)	11 359 (68.5)	14 753 (77.6)
Missing	371	112	110	91	237	146
No. of IV thrombolytic cases/y						
0-9	6682 (23.2)	1727 (21.8)	1669 (19.9)	1607 (23.0)	4195 (25.0)	3881 (20.3)
10-19	10 008 (34.7)	2823 (35.6)	2794 (33.3)	2764 (39.6)	5922 (35.3)	6664 (34.9)
20-29	7601 (26.4)	2091 (26.4)	2427 (28.9)	1690 (24.2)	4093 (24.4)	5274 (27.6)
30-126	4525 (15.7)	1278 (16.1)	1504 (17.9)	923 (13.2)	2544 (15.2)	3296 (17.2)
Missing	71	22	28	22	56	36
Teaching status						
Teaching	18 169 (68.1)	5060 (67.3)	5454 (68.0)	4297 (65.7)	9956 (64.3)	12 787 (71.9)
Missing	2207	421	401	465	1326	1359
Daily hospital census						
0-99	11 745 (44.0)	3221 (42.8)	3305 (41.2)	3408 (52.1)	7712 (49.8)	7238 (40.7)
100-199	9649 (36.2)	2848 (37.9)	3208 (40.0)	2347 (35.9)	5214 (33.7)	6687 (37.6)
≥200	5286 (19.8)	1451 (19.3)	1508 (18.8)	786 (12.0)	2558 (16.5)	3867 (21.7)
Missing	2207	421	401	465	1326	1359

Abbreviations: AIS, acute ischemic stroke; CAD, coronary artery disease; CT, computed tomography; ED, emergency department; EMS, emergency medical services; IV, intravenous; MI, myocardial infarction; NIHSS, National Institutes of Health Stroke Scale; PSC, primary stroke center; TIA, transient ischemic attack; VA, Veterans Affairs.

^a^ Indicates the use of interactive video-conferencing.

^b^ Includes American Indian or Alaska Native, Asian, Native Hawaiian or Other Pacific Islander, or unable to determine. Race and ethnicity were extracted from the medical record for inclusion in the registry.

^c^ Extracted from the medical record for inclusion in the registry. Patients with both Medicaid and Medicare were assigned to Medicare.

^d^ Results range from 0 to 42, with higher scores indicating greater stroke severity.

^e^ Included acute stroke–ready and noncertified hospitals.

### Statistical Analysis

Data were analyzed from July 8 to October 13, 2023. Generalized estimating equation (GEE) regression models were performed for DIDO time, controlling for a priori patient- and hospital-level characteristics. Next, each time was added separately to the model, and coefficients were compared among the 3 models (without door to imaging or imaging to door included, using door to imaging as a covariate, and using imaging to door as a covariate) available for DIDO time and process times while controlling for covariates. A similar analytic approach has been taken in prior projects examining the role of subintervals within larger phases of care in acute stroke and cardiovascular systems.^15,21^ We used GEE mean (rather than median) response models, given (1) partially attenuated skewness of residuals when assessing effects of time intervals on DIDO time, (2) ease of parameter interpretation, and (3) large sample size whereby the sampling distributions of parameter estimates are approximately normal, according to the central limit theorem.^22^ Contour plots were constructed for the joint distributions of DIDO and time variables using nonparametric kernel density estimation. A sensitivity analysis was conducted for door-to-imaging and imaging-to-door times whereby these process steps were treated as outcomes, and the effects of covariates were examined. Due to high missingness in times for door to emergency physician assessment (n = 18 670), door to initiation of vessel or perfusion imaging (n = 4065), door to telestroke activation (n = 21 318), door to stroke team activation (n = 20 419), and door to needle (n = 12 003), exploratory models were conducted to assess the associations of these intervals with DIDO time. All statistical tests were 2 sided, with an a priori level of significance set at α = .05. All statistical analyses were performed on the AHA Precision Medicine Platform using SAS Studio, version 9.4 (SAS Institute Inc), for data selection and initial data processing, and R, version 4.2.0, with R Studio, version 2022.07.01, for further data processing and analysis, using R markdown, version 2.16 (R Program for Statistical Computing). The R package geepack, version 1.3.9,^23^ was used to fit the GEE models, and R package MASS, version 7.3-56,^24^ was used to create the nonparametric contour plots.

## Results

A total of 28 887 patients with AIS transferred from 1595 hospitals were included in the primary analysis. Patients were 49.5% female and 50.5% male, with a mean (SD) age of 68.3 (14.8) years. In terms of race and ethnicity, 5.5% were Hispanic, 14.7% were non-Hispanic Black, 73.2% were White non-Hispanic, and 6.7% were non-Hispanic Other (including American Indian or Alaska Native, Asian, Native Hawaiian or Other Pacific Islander, or unable to determine). Most patients presented during the pandemic (60.4%) to urban (72.4%) and teaching (68.1%) hospitals, and most had prenotification by EMS (57.7%). The median NIHSS score was 10 (IQR, 5-18). Baseline characteristics are shown in Table 1. Most patients were transferred for consideration of EVT (n = 20 521), followed by postthrombolytic care (n = 10 940) and evaluation of thrombolytics (n = 465).

The mean (SD) DIDO time for patients was 171.4 (149.5) minutes, mean (SD) door-to-imaging time was 18.3 (34.1) minutes, and mean (SD) imaging-to-door time was 153.1 (141.5) minutes. A 1-minute increase in door-to-imaging time was associated with a 1.33 (95% CI, 1.07-1.59)–minute increase in the mean DIDO time, while a 1-minute increase in imaging-to-door time was associated with a 1.02 (95% CI, 1.01-1.03)–minute increase in mean DIDO time (Table 2).

**Table 2.** GEE Regression Results for the DTI and ITD Intervals Among 24 662 Patients

Characteristic	Model		
	Without DTI or ITD included, min (95% CI)	Using DTI as a covariate, min (95% CI)	Using ITD as a covariate, min (95% CI)
Intercept^a^	204.02	169.13	146.06
DTI	NA	1.33 (1.07 to 1.59)	NA
ITD	NA	NA	1.02 (1.01 to 1.03)
**Demographics**			
Age, y			
18-59	1 [Reference]	1 [Reference]	1 [Reference]
60-69	−4.41 (−9.32 to 0.51)	−2.94 (−7.60 to 1.71)	−1.00 (−2.47 to 0.47)
70-79	−0.94 (−6.41 to 4.53)	1.37 (−3.98 to 6.71)	−1.74 (−2.88 to −0.59)
80-110	4.94 (−0.19 to 10.07)	5.97 (1.02 to 10.92)	−0.86 (−2.18 to 0.47)
Sex			
Female	5.98 (2.13 to 9.83)	5.21 (1.55 to 8.87)	0.47 (−0.41 to 1.34)
Male	1 [Reference]	1 [Reference]	1 [Reference]
Race and ethnicity			
Hispanic	5.33 (−1.39 to 12.05)	1.54 (−4.63 to 7.71)	3.00 (0.63 to 5.36)
Non-Hispanic Black or African American	13.29 (7.10 to 19.48)	10.09 (4.21 to 15.96)	2.32 (1.09 to 3.56)
Non-Hispanic White	1 [Reference]	1 [Reference]	1 [Reference]
Non-Hispanic Other^b^	5.59 (−2.20 to 13.38)	5.74 (−1.84 to 13.32)	−0.06 (−1.46 to 1.34)
**Medical history**			
Hypertension	0.32 (−4.01 to 4.65)	−0.17 (−4.34 to 4.00)	0.33 (−0.50 to 1.15)
Dyslipidemia	−0.15 (−3.78 to 3.48)	−0.30 (−3.74 to 3.13)	0.18 (−0.79 to 1.15)
Diabetes	3.67 (−0.08 to 7.41)	3.18 (−0.33 to 6.70)	0.35 (−0.74 to 1.43)
Prior stroke	6.55 (1.78 to 11.31)	7.06 (2.39 to 11.73)	−0.46 (−1.35 to 0.42)
CAD or prior MI	0.34 (−4.21 to 4.90)	0.40 (−3.96 to 4.76)	−0.11 (−0.99 to 0.76)
Smoking	1.86 (−4.10 to 7.81)	0.90 (−4.79 to 6.60)	0.69 (−0.26 to 1.65)
Atrial fibrillation	−6.47 (−10.48 to −2.46)	−5.66 (−9.54 to −1.79)	−0.45 (−1.21 to 0.31)
Heart failure	0.71 (−4.80 to 6.22)	0.54 (−4.90 to 5.99)	0.16 (−0.79 to 1.11)
Prior TIA	−1.35 (−8.54 to 5.83)	−0.31 (−7.30 to 6.68)	−0.85 (−2.03 to 0.34)
Peripheral vascular disease	4.83 (−9.77 to 19.42)	5.62 (−8.70 to 19.94)	−0.49 (−2.12 to 1.14)
Carotid artery stenosis	5.89 (−4.83 to 16.61)	3.80 (−6.56 to 14.16)	1.41 (−0.17 to 3.00)
Prosthetic heart valve	−10.83 (−19.76 to −1.89)	−10.91 (−19.56 to −2.26)	−0.06 (−1.97 to 1.86)
Prior antithrombotic medication	−4.76 (−8.90 to −0.62)	−4.21 (−8.10 to −0.32)	−0.39 (−1.51 to 0.73)
**Arrival and clinical data**			
NIHSS score^c^			
0-6	1 [Reference]	1 [Reference]	1 [Reference]
7-12	−32.77 (−37.76 to −27.78)	−24.73 (−29.46 to −19.99)	−5.59 (−6.71 to −4.46)
13-19	−49.86 (−55.37 to −44.35)	−40.54 (−45.90 to −35.18)	−6.31 (−7.40 to −5.21)
≥20	−48.03 (−53.75 to −42.31)	−40.23 (−45.86 to −34.59)	−5.19 (−6.36 to −4.03)
Arrival mode and time			
Private	1 [Reference]	1 [Reference]	1 [Reference]
EMS no prenotification	4.26 (−2.12 to 10.63)	6.66 (0.60 to 12.71)	−2.09 (−3.80 to −0.39)
EMS prenotification	−15.60 (−20.97 to −10.24)	−2.87 (−8.44 to 2.70)	−9.35 (−10.77 to −7.93)
After hours	4.34 (0.86 to 7.83)	4.01 (0.70 to 7.32)	0.20 (−0.65 to 1.05)
During pandemic	7.38 (3.31 to 11.46)	7.18 (3.32 to 11.05)	0.08 (−0.95 to 1.12)
**Transferring hospital characteristics**			
Primary stroke center	−3.69 (−10.18 to 2.79)	−3.24 (−9.48 to 3.00)	−0.10 (−1.32 to 1.13)
Location			
Rural	1 [Reference]	1 [Reference]	1 [Reference]
Urban	−1.22 (−8.95 to 6.51)	−3.15 (−10.61 to 4.31)	1.64 (0.48 to 2.80)
Annual thrombolysis volume			
0-9	1 [Reference]	1 [Reference]	1 [Reference]
10-19	−4.28 (−12.35 to 3.79)	−2.96 (−10.74 to 4.82)	−0.94 (−2.46 to 0.57)
20-29	−7.91 (−18.06 to 2.25)	−6.77 (−16.63 to 3.10)	−0.87 (−2.58 to 0.84)
30-126	−8.18 (−19.94 to 3.57)	−5.42 (−16.59 to 5.75)	−1.79 (−3.93 to 0.35)
Teaching status			
Nonteaching	1 [Reference]	1 [Reference]	1 [Reference]
Teaching	−1.59 (−8.35 to 5.18)	−0.50 (−7.05 to 6.06)	−0.85 (−1.96 to 0.27)
Daily hospital census			
0-99	1 [Reference]	1 [Reference]	1 [Reference]
100-199	6.09 (−1.43 to 13.61)	3.26 (−4.11 to 10.63)	2.09 (0.84 to 3.35)
≥200	0.90 (−9.11 to 10.90)	−5.53 (−15.03 to 3.98)	4.53 (2.47 to 6.58)

Abbreviations: CAD, coronary artery disease; DTI, door to imaging; EMS, emergency medical services; GEE, generalized estimating equations; ITD, imaging to door; MI, myocardial infarction; NA, not applicable; NIHSS, National Institute of Health Stroke Scale; TIA, transient ischemic attack.

^a^ Represents the mean interval time for each model with all patient and hospital characteristics set as the reference category. Analysis outputs from these models are reported as minutes greater or less than the intercept (95% CI).

^b^ Includes American Indian or Alaska Native, Asian, Native Hawaiian or Other Pacific Islander, or unable to determine.

^c^ Results range from 0 to 42, with higher scores indicating greater stroke severity.

In the GEE model for DIDO with door-to-imaging time included as a covariate, additional factors associated with prolonged DIDO times were age of 80 years or older (compared with 18-59 years; 5.97 [95% CI, 1.02-10.92] minutes), female sex (5.21 [95% CI, 1.55-8.87] minutes), non-Hispanic Black race (compared with non-Hispanic White race; 10.09 [95% CI, 4.21-15.96] minutes), arrival during the COVID-19 pandemic (7.18 [95% CI, 3.32-11.05] minutes), EMS use without prenotification (compared with private arrival mode; 6.66 [95% CI, 0.60-12.71] minutes), after-hours arrival (4.01 [95% CI, 0.70-7.32] minutes), and history of stroke (7.06 [95% CI, 2.39-11.73]). Factors associated with reduced DIDO time were history of atrial fibrillation (−5.66 [95% CI, −9.54 to −1.79] minutes), history of prosthetic heart valve (−10.91 [95% CI, −19.56 to −2.26] minutes), prior antithrombotic medication use (−4.21 [95% CI, −8.10 to −0.32] minutes), and NIHSS score of 20 or greater (compared with a score of 0-6; −40.23 [95% CI, −45.86 to −34.59] minutes).

**Figure 3.**
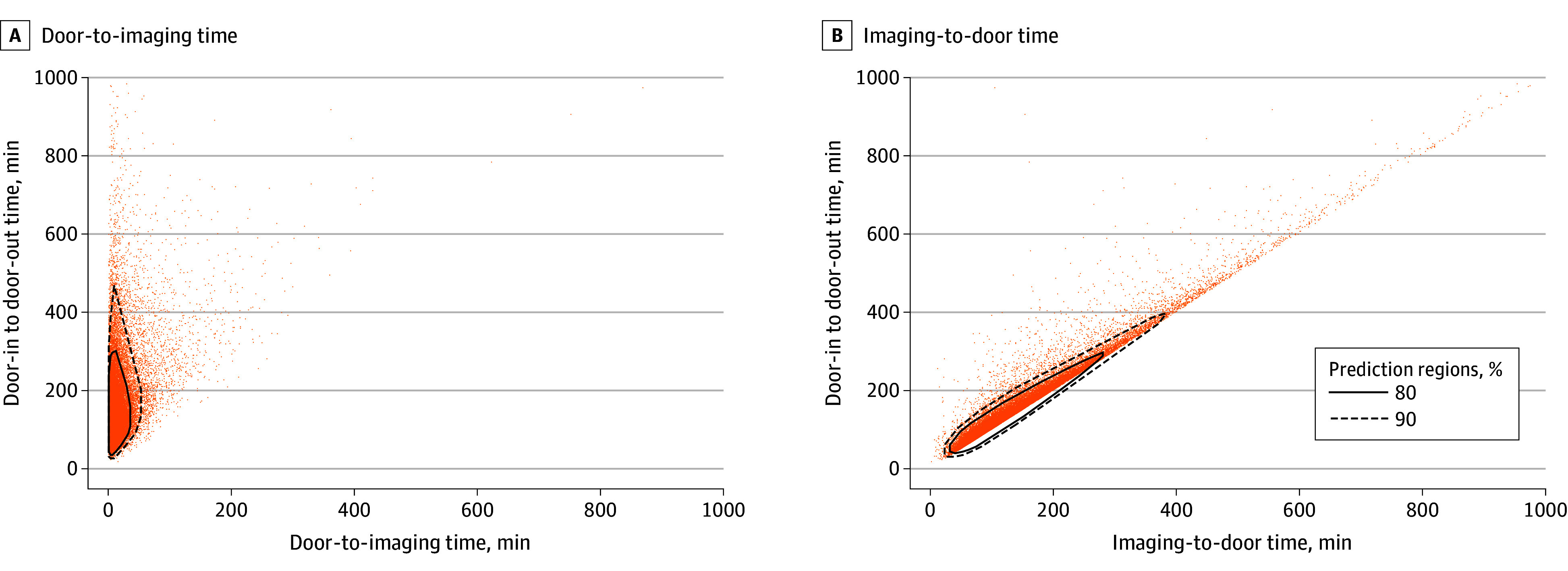
Contour Plots for Association Among Door-to-Imaging and Imaging-to-Door Times With Door-In–Door-Out Time

In the GEE model for DIDO with imaging-to-door time included, increasing age (≥80 years) and sex were no longer associated with prolonged DIDO time, but the following factors were: non-Hispanic Black race (compared with non-Hispanic White; 2.32 [95% CI, 1.09-3.56] minutes), Hispanic ethnicity (compared with non-Hispanic White; 3.00 [95% CI, 0.63-5.36] minutes), urban hospital location (1.64 [95% CI, 0.48-2.80] minutes), and daily hospital census of 200 patients or more (compared with 99 or fewer; 4.53 [95% CI, 2.47-6.58] minutes). The following were associated with reduced DIDO times: EMS prenotification (−9.35 [95% CI, −10.77 to −7.93] minutes) and NIHSS score of 20 or greater (compared with 0-6; −5.19 [95% CI, −6.36 to −4.03] minutes). Contour plots (at nominal levels of 80% and 90%) show the associations between door-to-imaging, imaging-to-door, and DIDO times and are displayed in Figure 3. In a sensitivity analysis with door-to-imaging and imaging-to-door intervals as outcomes, a similar direction of the findings was observed. Namely, age 80 years or older, female sex, and non-Hispanic Black race were associated with significantly prolonged imaging-to-door times, whereas age and sex became nonsignificant in the model with door-to-imaging time as the outcome, and the association for non-Hispanic Black race in this model was attenuated (eTable 1 in Supplement 1).

The mean (SD) time from door to emergency physician assessment was 8.8 (35.5) minutes, mean (SD) time from door to initiation of vessel or perfusion imaging was 46.0 (65.5) minutes, mean (SD) time from door to telestroke activation was 29.6 (39.1) minutes, mean (SD) time from door to stroke team activation was 5.8 (38.4) minutes, and mean (SD) door-to-needle time was 62.4 (40.5) minutes (eTables 2-6 in Supplement 1). Similar to imaging-to-door intervals that occurred after stroke diagnosis (emergency physician assessment to door, telestroke activation to door, stroke team activation to door, vessel or perfusion imaging to door, and needle to door) accounted for a greater proportion of DIDO.

## Discussion

Much attention and effort has been directed at expediting the assessment and treatment of patients with AIS when they present to a hospital; however, the results in this retrospective cohort study suggest that substantial delays occur when stroke care requires transfer to another hospital for treatment, primarily after imaging has been completed. In the present study, we found that the mean DIDO time was significantly prolonged at 171.4 minutes and the imaging-to-door time made up the vast majority of this interval (153.1 minutes), while the mean door-to-imaging time was 18.3 minutes.

Nationwide QI initiatives and guidelines have targeted aspects of early stroke care in the ED, such as door-to-imaging times.^13^ For instance, the AHA-ASA recommends a door-to-imaging time of 20 minutes.^13^ This threshold is currently being met in most cases within our study sample, which comprised patients potentially eligible for AIS treatment. This is encouraging, yet not surprising, given the substantial attention dedicated to this critical process metric and prior literature suggesting temporal improvements in door-to-imaging metrics.^14^ Stroke systems have implemented novel solutions to reduce door-to-imaging times—including the use of an all-points alarm to simultaneously alert all relevant staff to the arrival of a patient potentially eligible for thrombolysis—which have been associated with increased proportions of patients receiving thrombolysis.^25^ Similar to thrombolysis, the likelihood of good outcomes among patients receiving EVT is exquisitely time sensitive.^1,2^ In the EVT era, door-to-imaging times are still a key component of the acute stroke treatment framework; however, interhospital transfers represent an important contributor to treatment delays for EVT, and DIDO times have been an understudied area for stroke QI. Our findings suggest that further optimization and attention to the imaging-to-door subinterval of DIDO times is warranted.

Our results agree with the findings from several smaller studies^26,27^ that have shown that subinterval times occurring after initial brain imaging contribute more substantially to the overall DIDO time in acute stroke transfers. A retrospective cohort study in a Dutch ambulance region evaluated the determinants of DIDO time in 133 patients with AIS transferred for EVT and found that the time from computed tomographic angiography (CTA) to ambulance notification was the largest contributor (median, 24 [IQR, 16-37] minutes) to overall DIDO time.^26^ Another cohort study from Australia of 67 patients with AIS similarly found that CTA to ambulance notification represented the longest component (median, 59.5 [IQR, 44-83] minutes) of DIDO time.^27^ Though our study was unable to delineate time of ambulance notification, it is clear that the imaging-to-door interval represents a much greater proportion of DIDO time (approximately 89%) compared with door-to-imaging time (approximately 11%), and a major strength of the current study is the comparatively large sample size (28 887 patients with AIS).

Whereas the door-to-imaging period in the ED has received significant attention and standardization,^13^ the imaging-to-door period is complex, with multiple parallel processes involved. These tasks include interpretation of imaging, discussing results with the patient and family, informed consent process for treatment, and coordination of care and transfer logistics with outside EMS and hospital systems. Additionally, such processes are potentially subject to substantial intrahospital and interhospital variation.^27^ However, the fact that imaging-to-door time made up such a large proportion and explained most of the variance in overall DIDO times in this nationally representative sample from the GWTG-Stroke registry suggests that there is a dire need for improvement in this complex process, and there are likely best practices that can be identified and disseminated to improve patient care across the nation.

Disparities in the imaging-to-door period also largely explained the disparities in DIDO time by non-Hispanic Black race, older age, and female sex. In the GEE model including door-to-imaging time as a covariate (but not imaging-to-door time), age 80 years or older, female sex, and non-Hispanic Black race were all associated with longer DIDO times, similar to the inequities demonstrated in the prior study of DIDO times using the GWTG-Stroke registry.^7^ However, in the GEE model adjusting for imaging-to-door time, there were no significant disparities by age 80 years or older or female sex, and the disparity by non-Hispanic Black race was attenuated. The fact that DIDO disparities are attenuated by adding the imaging-to-door period into the model reflects that the underlying disparities in DIDO are driven by disparities in imaging-to-door times. These findings suggest that future systems of care interventions should target reducing overall and specifically disparities in imaging-to-door time.

To determine whether similar findings were present in other acute process steps, we explored the relative contributions of the before and after subintervals for the following key process steps in the management of AIS: ED physician assessment, telestroke or stroke team activation, vessel or perfusion imaging, and thrombolysis administration. The delay in time from door to initiation of vessel or perfusion imaging time (46.0 minutes vs 18.3 minutes for door to initial imaging time) suggests a portion of patients are returning for vessel and perfusion imaging rather than obtaining bundled CT, CTA, and CT perfusion imaging. This finding aligns with a prior study of DIDO process times at primary stroke centers in Chicago,^6^ which found that delays in obtaining CTA were a strong driver of prolonged DIDO times. These findings provide further evidence that bundled CT imaging up front, when warranted, is likely an important modifiable system redesign to improve transfer times. However, it is worth noting that among those eligible for thrombolytic therapy, it may be preferable to initiate thrombolysis immediately following non–contrast-enhanced head CT, rather than a strict bundled imaging approach depending on local protocols and practices.^28^ The mean time from door to emergency physician assessment was 8.8 minutes (compared to a guideline-recommended goal of ≤10 minutes)^13^; mean time from door to stroke team contact was 5.8 minutes (goal, ≤15 minutes)^13^; and mean door-to-needle time was 62.4 minutes (goal, ≤60 minutes). These findings suggest that patients with AIS eligible for acute treatment were treated expeditiously and largely within guideline-recommended targets.^13^ Similar to the findings for the primary analysis, the subintervals in the latter half of the overall DIDO equation accounted for a greater proportion of variation in DIDO times. These findings from the exploratory analyses further support the findings from the primary analysis and suggest that while existing guidelines and care resources heavily focus on the initial acute phase of stroke process metrics, future QI efforts should focus on expediting the steps after imaging to achieve DIDO time goals for interhospital transfers of patients with AIS.

An area of particular focus for future research should include characterizing delays in request and availability of interfacility transport by EMS or a third party to the receiving hospital. Determining what contributions to delays come from hospital practice (eg, delays in requesting transport) or from EMS (eg, availability of suitable transport) could help focus additional interventions to reduce DIDO time. This work would be aided by the addition of these variables to local and national QI stroke registries.

### Limitations

This study is limited by incomplete and missing data from transferring hospitals. Additionally, we did not capture data from non–GWTG-participating hospitals, which may skew these data to more resourced hospitals that participate in QI measures. Next, some potential contributors to DIDO time were not included in this analysis such as simultaneous vs consecutive vascular imaging (though not explicitly included as a separate variable, the time to vessel or perfusion imaging was much longer on average, suggesting many sites do not bundle this imaging up front). The study period also includes the COVID-19 pandemic, which may have unmeasured effects on the length of DIDO times. Previous studies have shown that limited bed availability, especially early in the pandemic, was a factor in decreased thrombectomy rates in France,^29^ while a multicenter study in the US found no delay in EVT during the COVID-19 period compared with the pre–COVID-19 period.^30^

## Conclusions

In this retrospective cohort study of a national, hospital-based registry of patients with AIS, multiple process steps contributed to delays in interhospital transfer. Future QI efforts and stroke care guidelines should focus on identifying and disseminating best practices for management after imaging to expedite the transfer of patients with AIS and ensure the best outcome possible.

## Notes

Supplement 1. **eTable 1.** GEE Regression Results for the Door-to-Imaging (DTI) and Imaging-to-Door (ITD) Intervals as Outcomes **eTable 2.** GEE Regression Results for the Door-to–Emergency Physician Evaluation (DTEP) and Emergency Physician Evaluation–to-Door (EPTD) Intervals **eTable 3.** GEE Regression Results for the Door-to–Stroke Team Activation (DTST) and Stroke Team Activation–to-Door (STTD) Intervals **eTable 4.** GEE Regression Results for the Door-to–Telestroke Activation (DTT) and Telestroke Activation–to-Door (TTD) Intervals **eTable 5.** GEE Regression Results for the Door-to–Vessel or Perfusion Imaging (DTVP) and Vessel or Perfusion Imaging–to-Door (VPTD) Intervals **eTable 6.** GEE Regression Results for the Door-to-Thrombolysis (Needle) (DTN) and Thrombolysis (Needle)-to-Door (NTD) Intervals

Supplement 2. Data Sharing Statement
