# Supplementary material for: Emergency Department Process Times and Door-In–Door-Out Times in Interhospital Transfers After Acute Ischemic Stroke
Source: JAMA Netw Open. 2024 Sep 3;7(9):e2431183. doi: 10.1001/jamanetworkopen.2024.31183 (PMC11372507; doi:10.1001/jamanetworkopen.2024.31183)
Supplement: Supplement 1. — eTable 1. GEE Regression Results for the Door-to-Imaging (DTI) and Imaging-to-Door (ITD) Intervals as Outcomes eTable 2. GEE Regression Results for the Door-to–Emergency Physician Evaluation (DTEP) and Emergency Physician Evaluation–to-Door (EPTD) Intervals eTable 3. GEE Regression Results for the Door-to–Stroke Team Activation (DTST) and Stroke Team Activation–to-Door (STTD) Intervals eTable 4. GEE Regression Results for the Door-to–Telestroke Activation (DTT) and Telestroke Activation–to-Door (TTD) Intervals eTable 5. GEE Regression Results for the Door-to–Vessel or Perfusion Imaging (DTVP) and Vessel or Perfusion Imaging–to-Door (VPTD) Intervals eTable 6. GEE Regression Results for the Door-to-Thrombolysis (Needle) (DTN) and Thrombolysis (Needle)-to-Door (NTD) Intervals [file jamanetwopen-e2431183-s001.pdf]

Supplementary Online Content

Royan R, Stamm B, Giurcanu M, Messe SR, Jauch EC, Prabhakaran S. Door-in to door-out times in interhospital transfers of patients with acute ischemic stroke. *JAMA Netw Open*. 2024;7(9):e2431183. doi:10.1001/jamanetworkopen.2024.31183

eTable 1. GEE Regression Results for the Door-to-Imaging (DTI) and Imaging-to-Door (ITD) Intervals as Outcomes

eTable 2. GEE Regression Results for the Door-to–Emergency Physician Evaluation (DTEP) and Emergency Physician Evaluation–to-Door (EPTD) Intervals

eTable 3. GEE Regression Results for the Door-to–Stroke Team Activation (DTST) and Stroke Team Activation–to-Door (STTD) Intervals

eTable 4. GEE Regression Results for the Door-to–Telestroke Activation (DTT) and Telestroke Activation–to-Door (TTD) Intervals

eTable 5. GEE Regression Results for the Door-to–Vessel or Perfusion Imaging (DTVP) and Vessel or Perfusion Imaging–to-Door (VPTD) Intervals

eTable 6. GEE Regression Results for the Door-to-Thrombolysis (Needle) (DTN) and Thrombolysis (Needle)-to-Door (NTD) Intervals

This supplementary material has been provided by the authors to give readers additional information about their work.

© 2024 Royan R et al. *JAMA Network Open*.

eTable 1. GEE Regression Results for the Door-to-Imaging (DTI) and Imaging-to-Door (ITD) Intervals as Outcomes

	Door-to-Imaging as Outcome Minutes (95% CI)	Imaging-to-Door as Outcome Minutes (95% CI)
**Intercept**[a]	26.15 (23.64, 28.67)	177.76 (166.57, 188.94)
**DEMOGRAPHICS**		
**Age (Years)**		
18-59	Ref	Ref
60-69	-1.06 (-2.54, 0.42)	-3.31 (-7.90, 1.29)
70-79	-1.72 (-2.86, -0.58)	-0.80 (-4.55, 6.15)
80-110	-0.76 (-2.07, 0.55)	5.72 (0.77, 10.67)
**Sex**		
Male	Ref	Ref
Female	0.57 (-0.30, 1.44)	5.40 (1.72, 9.08)
**Race/Ethnicity**		
Black or African American non-Hispanic	2.60 (1.35, 3.84)	10.85 (4.97, 16.72)
Hispanic	3.11 (0.74, 5.47)	2.44 (-3.68, 8.56)
Other non-Hispanic[b]	0.05 (-1.34, 1.44)	5.70 (-1.89, 13.28)
White non-Hispanic	Ref	Ref
**Medical History / Prior Meds**		
Hypertension	0.34 (-0.49, 1.16)	-0.06 (-4.24, 4.13)
Dyslipidemia	0.16 (-0.82, 1.13)	-0.26 (-3.70, 3.19)
Diabetes	0.42 (-0.67, 1.50)	3.30 (-0.20, 6.80)
Prior stroke	-0.33 (-1.21, 0.55)	6.93 (2.26, 11.60)
CAD / Prior MI	-0.09 (-0.97, 0.78)	0.38 (-4.00, 4.76)
Smoking	0.71 (-0.26, 1.68)	1.14 (-4.59, 6.87)
Atrial fibrillation	-0.56 (-1.32, 0.20)	-5.86 (-9.76, -1.97)
Heart failure	0.17 (-0.77, 1.11)	0.58 (-4.85, 6.02)
Prior TIA	-0.87 (-2.05, 0.32)	-0.56 (-7.57, 6.45)
Peripheral vascular disease	-0.40 (-2.03, 1.23)	5.43 (-8.92, 19.79)
Carotid artery stenosis	1.52 (-0.09, 3.12)	4.31 (-6.11, 14.72)
Prosthetic heart valve	-0.28 (-2.20, 1.64)	-10.88 (-19.54, -2.23)
Prior antithrombotic medication	-0.46 (-1.58, 0.65)	-4.35 (-8.25, -0.45)
**ARRIVAL & CLINICAL DATA**		
**NIH Stroke Scale Score**[c]		
0-6	Ref	Ref
7-12	-6.10 (-7.29, -4.92)	-26.72 (-31.43, -22.00)
13-19	-7.13 (-8.32, -5.94)	-42.85 (-48.05, -37.65)
≥20	-5.99 (-7.20, -4.78)	-42.17 (-47.57, -36.77)
**Arrival Mode / Time**		
Private arrival mode	Ref	Ref
EMS no pre-notification	-1.97 (-3.68, -0.26)	6.06 (-0.00, 12.12)
EMS pre-notification	-9.44 (-10.86, -8.03)	-6.03 (-11.03, -1.02)
**After Hours**	0.28 (-0.57, 1.14)	4.09 (0.78, 7.41)
**During Pandemic**	0.23 (-0.81, 1.26)	7.23 (3.37, 11.09)
**TRANSFERRING HOSPITAL CHARACTERISTICS**		
**Primary Stroke Center**	-0.20 (-1.43, 1.03)	-3.33 (-9.59, 2.93)
**Location**		

© 2024 Royan R et al. *JAMA Network Open*.

Rural	Ref	Ref
Urban	1.59 (0.42, 2.75)	-2.67 (-10.17, 4.83)
**Annual Thrombolysis Volume**		
0-9	Ref	Ref
10-19	-1.03 (-2.54, 0.48)	-3.28 (-11.10, 4.55)
20-29	-1.01 (-2.71, 0.69)	-7.05 (-16.95, 2.85)
30-126	-1.94 (-4.09, 0.21)	-6.09 (-17.32, 5.14)
**Teaching Status**		
Non-Teaching	Ref	Ref
Teaching	-0.85 (-1.96, 0.26)	-0.77 (-7.36, 5.81)
**Daily Hospital Census**		
0-99	Ref	Ref
100-199	2.16 (0.90, 3.41)	3.97 (-3.36, 11.29)
≥200	4.42 (2.37, 6.46)	-3.92 (-13.36, 5.52)

CI=Confidence Interval; CAD=Coronary Artery Disease; MI=Myocardial Infarction; TIA=Transient Ischemic Attack; NIH=National Institute of Health; EMS=Emergency Medical System

a. The intercept represents the mean interval time for each model with all patient and hospital characteristics set as the reference category. Analysis outputs from these models are reported as minutes greater/less than the intercept.
b. Other includes American Indian/Alaska Native, Asian, Native Hawaiian or Pacific Islander, or Unable to Determine. Race and ethnicity were extracted from the medical record for inclusion in the registry.
c. National Institutes of Health Stroke Scale (NIHSS) results range from 0-42, with higher scores indicating greater stroke severity.

© 2024 Royan R et al. *JAMA Network Open*.

eTable 2. GEE Regression Results for the Door-to–Emergency Physician Evaluation (DTEP) and Emergency Physician Evaluation–to-Door (EPTD) Intervals

	Model 1 (without DTEP or EPTD included) Minutes (95% CI)	Model 2 (using DTEP as a covariate) Minutes (95% CI)	Model 3 (using EPTD as a covariate) Minutes (95% CI)
**Intercept**[a]	193.83 (173.91, 213.74)	179.62 (161.24, 198.01)	144.35 (137.88, 150.82)
**Door to Emergency Physician Evaluation (DTEP)**	—	1.19 (0.85, 1.53)	—
**Emergency Physician Evaluation to Door (EPTD)**	—	—	1.02 (1.00, 1.03)
**DEMOGRAPHICS**			
**Age (Years)**			
18-59	Ref	Ref	Ref
60-69	-4.32 (-14.31, 5.67)	-5.33 (-14.50, 3.84)	0.94 (-2.35, 4.22)
70-79	-5.59 (-13.17, 1.98)	-4.47 (-11.97, 3.03)	-0.82 (1.99, 0.35)
80-110	1.50 (-8.83, 11.84)	2.50 (-7.68, 12.68)	-0.92 (-2.43, 0.59)
**Sex**			
Male	Ref	Ref	Ref
Female	7.36 (0.85, 13.88)	5.69 (-0.44, 11.81)	1.49 (-0.55, 3.53)
**Race/Ethnicity**			
Black or African American non-Hispanic	9.05 (-1.32, 19.42)	9.52 (-0.68, 19.72)	-0.07 (-1.41, 1.27)
Hispanic	11.84 (-1.06, 24.74)	10.71 (-1.92, 23.34)	0.27 (-1.82, 2.36)
Other non-Hispanic[b]	8.11 (-6.24, 22.46)	8.73 (-5.16, 22.62)	-0.64 (-2.66, 1.38)
White non-Hispanic	Ref	Ref	Ref
**Medical History / Prior Meds**			
Hypertension	1.86 (-7.30, 11.02)	4.69 (-3.38, 12.76)	-2.52 (-7.18, 2.13)
Dyslipidemia	-1.76 (-8.32, 4.81)	-2.95 (-8.92, 3.03)	0.85 (-1.17, 2.88)
Diabetes	8.43 (1.55, 15.30)	6.15 (-0.33, 12.63)	2.07 (-0.50, 4.64)
Prior stroke	4.65 (-3.61, 12.91)	2.94 (-4.16, 10.04)	1.31 (-2.60, 5.22)
CAD / Prior MI	0.01 (-7.19, 7.19)	-1.81 (-8.39, 4.76)	1.82 (-1.06, 4.70)
Smoking	0.29 (-11.76, 12.34)	1.87 (-9.99, 13.72)	-1.13 (-3.03, 0.77)
Atrial fibrillation	-0.14 (-8.89, 8.60)	0.55 (-7.66, 8.75)	-0.60 (-3.40, 2.19)
Heart failure	3.30 (-8.95, 15.55)	-2.13 (-10.77, 6.51)	4.58 (-4.26, 13.41)
Prior TIA	-0.55 (-10.03, 8.93)	1.83 (-7.27, 10.93)	-2.06 (-4.56, 0.44)
Peripheral vascular disease	-6.64 (-21.71, 8.43)	-4.39 (-19.12, 10.34)	-2.00 (-5.52, 1.52)
Carotid artery stenosis	-9.85 (-20.56, 0.86)	-6.59 (-16.56, 3.38)	-2.48 (-5.52, 1.52)
Prosthetic heart valve	-10.19 (-28.96, 8.57)	-9.58 (-28.60, 9.45)	-1.02 (-4.43, 2.39)
Prior antithrombotic medication	-2.49 (-10.09, 5.10)	-2.43 (-9.70, 4.85)	-0.04 (-1.22, 1.13)
**ARRIVAL & CLINICAL DATA**			
**NIH Stroke Scale Score**[c]			
0-6	Ref	Ref	Ref
7-12	-26.24 (-34.66, -17.82)	-23.95 (-32.12, -15.78)	-1.28 (-2.45, -0.11)
13-19	-42.56 (-50.99, -34.13)	-41.58 (-49.11, -34.05)	0.11 (-3.70, 3.92)
≥20	-39.36 (-49.82, -28.90)	-36.51 (-46.65, -26.38)	-1.79 (-3.17, -0.42)
**Arrival Mode / Time**			
Private arrival mode	Ref	Ref	Ref
EMS no pre-notification	7.48 (-4.94, 19.91)	10.65 (-1.68, 22.97)	-2.62 (-3.89, -1.34)
EMS pre-notification	-8.78 (-17.12, -0.45)	-4.84 (-12.81, 3.14)	-3.03 (-5.03, -1.02)

© 2024 Royan R et al. *JAMA Network Open*.

**After Hours**	4.57 (-1.32, 10.46)	5.09 (-0.52, 10.71)	-0.62 (-2.35, 1.11)
**During Pandemic**	12.80 (6.40, 19.20)	12.65 (6.29, 19.01)	-0.03 (-1.80, 1.75)
**TRANSFERRING HOSPITAL CHARACTERISTICS**			
**Primary Stroke Center**	-3.25 (-14.16, 7.66)	-4.25 (-13.98, 5.48)	0.53 (-2.96, 4.02)
**Location**			
Rural	Ref	Ref	Ref
Urban	-7.29 (-21.33, 6.75)	-3.78 (-16.59, 9.02)	-2.50 (-6.71, 1.71)
**Annual Thrombolysis Volume**			
0-9	Ref	Ref	Ref
10-19	4.43 (-9.47, 18.32)	6.85 (-5.69, 19.40)	-1.86 (-6.92, 3.19)
20-29	-8.24 (-23.96, 7.47)	-5.24 (-19.51, 9.02)	-1.90 (-6.39, 2.59)
30-126	-3.13 (-22.93, 16.68)	-1.50 (-20.35, 17.36)	-1.18 (-5.20, 2.83)
**Teaching Status**			
Non-Teaching	Ref	Ref	Ref
Teaching	-9.55 (-21.56, 2.45)	-5.68 (-16.58, 5.23)	-2.45 (-5.64, 0.74)
**Daily Hospital Census**			
0-99	Ref	Ref	Ref
100-199	-4.62 (-16.89, 7.65)	-3.54 (-15.12, 8.04)	-0.85 (-3.03, 1.33)
≥200	1.79 (-16.28, 19.87)	2.11 (-15.17, 19.39)	-0.64 (-2.41, 1.14)

CI=Confidence Interval; CAD=Coronary Artery Disease; MI=Myocardial Infarction; TIA=Transient Ischemic Attack; NIH=National Institute of Health; EMS=Emergency Medical System

a. The intercept represents the mean interval time for each model with all patient and hospital characteristics set as the reference category. Analysis outputs from these models are reported as minutes greater/less than the intercept.

b. Other includes American Indian/Alaska Native, Asian, Native Hawaiian or Pacific Islander, or Unable to Determine. Race and ethnicity were extracted from the medical record for inclusion in the registry.

c. National Institutes of Health Stroke Scale (NIHSS) results range from 0-42, with higher scores indicating greater stroke severity.

© 2024 Royan R et al. *JAMA Network Open*.

eTable 3. GEE Regression Results for the Door-to–Stroke Team Activation (DTST) and Stroke Team Activation–to-Door (STTD) Intervals

	Model 1 (without DTST or STTD included) Minutes (95% CI)	Model 2 (using DTST as a covariate) Minutes (95% CI)	Model 3 (using STTD as a covariate) Minutes (95% CI)
Intercept[a]	176.76 (159.07, 194.45)	162.50 (145.24, 179.76)	141.33 (133.14, 149.52)
**Door to Stroke Team Activation (DTST)**	—	1.09 (0.90, 1.27)	—
**Stroke Team Activation to Door (STTD)**	—	—	1.00 (0.98, 1.02)
**DEMOGRAPHICS**			
**Age (Years)**			
18-59	Ref	Ref	Ref
60-69	-0.70 (-9.14, 7.75)	-0.38 (-7.98, 7.22)	0.72 (-4.01, 5.45)
70-79	0.56 (-6.64, 7.81)	2.00 (-5.19, 9.19)	-2.29 (-6.00, 1.43)
80-110	6.10 (-3.63, 15.83)	7.12 (-2.39, 16.63)	1.41 (-2.49, 5.31)
**Sex**			
Male	Ref	Ref	Ref
Female	9.31 (3.22, 15.40)	7.68 (2.12, 13.23)	2.61 (-0.46, 5.67)
**Race/Ethnicity**			
Black or African American non-Hispanic	11.43 (1.92, 20.94)	10.76 (1.39, 20.13)	1.28 (-2.59, 5.13)
Hispanic	16.99 (5.48, 28.50)	14.28 (3.13, 25.43)	3.14 (-2.81, 9.10)
Other non-Hispanic[b]	10.27 (-1.03, 21.57)	9.28 (-0.75, 19.31)	2.72 (-3.65, 9.09)
White non-Hispanic	Ref	Ref	Ref
**Medical History / Prior Meds**			
Hypertension	2.88 (-5.45, 11.22)	6.23 (-0.96, 13.42)	-2.54 (-7.37, 2.29)
Dyslipidemia	-1.46, (-6.87, 3.95)	-3.57 (-8.63, 1.50)	1.65 (-1.70, 5.00)
Diabetes	2.89 (-3.40, 9.18)	0.76 (-5.07, 6.59)	3.21 (-0.48, 6.91)
Prior stroke	8.41 (0.21, 16.60)	6.32 (-0.71, 13.35)	5.16 (-0.52, 10.83)
CAD / Prior MI	4.01 (-3.25, 11.26)	1.91 (-4.87, 8.69)	2.93 (-1.35, 7.21)
Smoking	1.67 (-10.37, 13.71)	2.85 (-8.65, 14.34)	-2.37 (-5.97, 1.22)
Atrial fibrillation	-2.87 (-10.60, 4.85)	-1.79 (-8.93, 5.36)	-0.30 (-4.17, 3.56)
Heart failure	0.98 (-10.85, 12.81)	-3.36 (-11.45, 4.73)	8.07 (-2.79, 18.93)
Prior TIA	7.85 (-4.01, 19.71)	8.77 (-2.81, 20.35)	0.78 (-4.41, 5.98)
Peripheral vascular disease	6.18 (-9.02, 21.38)	7.90 (-6.67, 22.46)	-2.70 (-8.42, 3.75)
Carotid artery stenosis	-5.03 (-16.04, 5.98)	-3.72 (-14.03, 6.59)	-2.36 (-8.47, 3.75)
Prosthetic heart valve	-16.50, (-29.68, -3.33)	-13.34 (-26.85, 0.17)	-3.00 (-11.82, 5.83)
Prior antithrombotic medication	-1.32 (-7.44, 4.80)	-0.96 (-6.88, 4.96)	-0.24 (-3.04, 2.56)
**ARRIVAL & CLINICAL DATA**			
**Activation of Stroke Team Prior to Arrival**	—	-14.22 (-20.92, -7.51)	9.16 (1.20, 17.12)
**NIH Stroke Scale Score[c]**			
0-6	Ref	Ref	Ref
7-12	-26.73 (-34.88, -18.58)	-22.47 (-30.35, -14.59)	-5.40 (-8.79, -2.00)
13-19	-39.74 (-47.26, -32.21)	-36.37 (-43.20, -29.53)	-7.73 (-12.14, -3.31)
≥20	-38.33 (-47.50, -29.17)	-33.86 (-42.93, -24.79)	-9.94 (-13.73, -6.14)
**Arrival Mode / Time**			
Private arrival mode	Ref	Ref	Ref
EMS no pre-notification	10.23 (-1.49, 21.95)	11.35 (-0.29, 22.99)	1.82 (-1.79, 5.44)
EMS pre-notification	-7.89 (-15.43, -0.33)	1.04 (-6.49, 8.56)	-2.52 (-6.48, 1.44)
**After Hours**	5.41 (-0.06, 10.88)	5.97 (0.81, 11.13)	0.89 (-1.89, 3.66)

© 2024 Royan R et al. *JAMA Network Open*.

**During Pandemic**	8.96 (2.56, 15.37)	8.35 (2.20, 14.51)	0.87 (-2.24, 3.97)
**TRANSFERRING HOSPITAL CHARACTERISTICS**			
**Primary Stroke Center**	5.70 (-4.24, 15.63)	5.49 (-4.04, 15.02)	3.63 (-1.92, 9.17)
**Location**			
Rural	Ref	Ref	Ref
Urban	-0.29 (-16.06, 11.49)	0.53 (-11.58, 12.64)	0.10 (-6.65, 6.84)
**Annual Thrombolysis Volume**			
0-9	Ref	Ref	Ref
10-19	-2.29 (-16.06, 11.49)	1.66 (-11.43, 14.74)	-1.23 (-8.32, 5.86)
20-29	-7.94 (-23.81, 7.92)	-4.08 (-19.35, 11.19)	-5.62 (-12.92, 1.69)
30-126	-11.28 (-28.03, 5.47)	-7.02 (-23.88, 9.84)	-2.71 (-10.53, 5.11)
**Teaching Status**			
Non-Teaching	Ref	Ref	Ref
Teaching	-2.94 (-14.80, 8.91)	-0.11 (-11.51, 11.29)	-1.68 (-7.69, 4.32)
**Daily Hospital Census**			
0-99	Ref	Ref	Ref
100-199	-9.95 (-20.78, 0.89)	-8.41 (-19.22, 2.39)	-3.40 (-8.54, 1.73)
≥200	-1.71 (-17.76, 14.34)	-4.12 (-19.53, 11.29)	1.37 (-5.69, 8.43)

CI=Confidence Interval; CAD=Coronary Artery Disease; MI=Myocardial Infarction; TIA=Transient Ischemic Attack; NIH=National Institute of Health; EMS=Emergency Medical System

a. The intercept represents the mean interval time for each model with all patient and hospital characteristics set as the reference category. Analysis outputs from these models are reported as minutes greater/less than the intercept.
b. Other includes American Indian/Alaska Native, Asian, Native Hawaiian or Pacific Islander, or Unable to Determine. Race and ethnicity were extracted from the medical record for inclusion in the registry.
c. National Institutes of Health Stroke Scale (NIHSS) results range from 0-42, with higher scores indicating greater stroke severity.

© 2024 Royan R et al. *JAMA Network Open*.

eTable 4. GEE Regression Results for the Door-to–Telestroke Activation (DTT) and Telestroke Activation–to-Door (TTD) Intervals

	**Model 1 (without DTT or TTD included) Minutes (95% CI)**	**Model 2 (using DTT as a covariate) Minutes (95% CI)**	**Model 3 (using TTD as a covariate) Minutes (95% CI)**
Intercept[a]	198.61 (180.62, 216.59)	171.48 (154.38, 188.59)	160.73 (154.92, 166.53)
**Door to Telestroke Activation (DTT)**	—	0.98 (0.83, 1.14)	—
**Telestroke Activation to Door (TTD)**	—	—	1.00 (0.98, 1.01)
**DEMOGRAPHICS**			
**Age (Years)**			
18-59	Ref	Ref	Ref
60-69	-5.28 (-12.35, 1.78)	-4.23 (-10.69, 2.23)	-1.12 (-3.82, 1.59)
70-79	8.56 (-2.00, 19.13)	10.32 (-0.02, 20.66)	-1.08 (-4.22, 2.07)
80-110	12.95 (2.15, 23.74)	11.95 (0.88, 23.02)	1.22 (-2.01, 4.44)
**Sex**			
Male	Ref	Ref	Ref
Female	3.48 (-2.52, 9.48)	2.12 (-3.62, 7.86)	1.73 (-0.23, 3.68)
**Race/Ethnicity**			
Black or African American non-Hispanic	5.92 (-4.55, 16.39)	5.10 (-5.24, 15.45)	0.93 (-2.16, 4.03)
Hispanic	-1.96 (-14.81, 10.89)	-2.14 (-14.31, 10.03)	-0.85 (-5.82, 4.13)
Other non-Hispanic[b]	12.08 (-10.55, 34.71)	10.33 (-11.38, 32.04)	0.77 (-3.80, 5.34)
White non-Hispanic	Ref	Ref	Ref
**Medical History / Prior Meds**			
Hypertension	0.80 (-8.79, 10.38)	1.23 (-8.23, 10.69)	-0.36 (-2.84, 2.12)
Dyslipidemia	2.12 (-4.96, 9.20)	2.00 (-4.88, 8.88)	0.16 (-1.98, 2.29)
Diabetes	0.40 (-6.50, 7.30)	-1.11 (-7.44, 5.22)	1.29 (-1.04, 3.61)
Prior stroke	5.22 (-4.29, 14.72)	4.11 (-5.25, 13.47)	1.10 (-1.51, 3.70)
CAD / Prior MI	4.98 (-6.01, 15.98)	5.64 (-5.49, 16.77)	-0.40 (-2.83, 2.04)
Smoking	4.40 (-8.32, 17.12)	1.59 (-11.16, 14.33)	3.38 (0.19, 6.57)
Atrial fibrillation	-9.32 (-17.49, -1.14)	-9.12 (-17.04, -1.20)	-1.53 (-4.09, 1.02)
Heart failure	1.87 (-7.46, 11.20)	1.40 (-7.87, 10.67)	-0.05 (-3.44, 3.34)
Prior TIA	-2.44 (-12.54, 7.66)	-0.57 (-10.70, 9.55)	-1.98 (-5.14, 1.18)
Peripheral vascular disease	-5.43 (-23.05, 12.20)	-7.63 (-24.16, 8.90)	2.81 (-4.96, 10.58)
Carotid artery stenosis	11.19 (-16.37, 38.74)	10.53 (-16.84, 37.90)	0.70 (-5.64, 7.04)
Prosthetic heart valve	-5.56 (-23.37, 12.24)	-8.41 (-25.95, 9.12)	0.87 (-6.49, 8.23)
Prior antithrombotic medication	-9.19 (-18.52, 0.13)	-9.15 (-18.20, -0.09)	-0.33 (-2.73, 2.07)
**ARRIVAL & CLINICAL DATA**			
**Activation of Telestroke Team Prior to Arrival**	—	-18.12 (-29.37, -6.88)	-3.47 (-17.82, 10.88)
**NIH Stroke Scale Score[c]**			
0-6	Ref	Ref	Ref
7-12	-23.63 (-34.36, -12.90)	-16.40 (-26.92, -5.88)	-7.39 (-10.62, -4.16)
13-19	-46.71 (-55.24, -38.19)	-38.56 (-47.08, -30.03)	-9.00 (-12.05, -5.96)
≥20	-44.73 (-54.93, -34.54)	-36.60 (-47.05, -26.15)	-9.36 (-12.63, -6.09)
**Arrival Mode / Time**			

© 2024 Royan R et al. *JAMA Network Open*.

Private arrival mode	Ref	Ref	Ref
EMS no pre-notification	6.45 (-6.39, 19.28)	6.16 (-6.12, 18.44)	0.71 (-3.21, 4.63)
EMS pre-notification	-18.51 (-27.15, -9.88)	-9.82 (-17.90, -1.75)	-7.10 (-10.53, -3.68)
**After Hours**	3.60 (-3.09, 10.28)	4.73 (-1.80, 11.26)	-0.83 (-2.77, 1.10)
**During Pandemic**	6.73 (-0.63, 14.09)	14.15 (7.01, 21.28)	-6.60 (-9.23, -3.97)
**TRANSFERRING HOSPITAL CHARACTERISTICS**			
**Primary Stroke Center**	-1.43 (-13.54, 10.68)	-3.22 (-14.25, 7.80)	1.65 (-2.64, 5.94)
**Location**			
Rural	Ref	Ref	Ref
Urban	-3.31 (-15.69, 9.08)	-0.26 (-11.95, 11.43)	-2.70 (-7.24, 1.84)
**Annual Thrombolysis Volume**			
0-9	Ref	Ref	Ref
10-19	-3.63 (-16.03, 8.76)	-2.66 (-14.54, 9.22)	0.10 (-4.21, 4.42)
20-29	15.68 (-4.17, 35.53)	17.67 (-0.38, 35.71)	-2.36 (-8.27, 3.55)
30-126	-1.05 (-26.65, 24.56)	5.66 (-16.56, 27.89)	-7.92 (-17.06, 1.23)
**Teaching Status**			
Non-Teaching	Ref	Ref	Ref
Teaching	0.78 (-11.46, 13.03)	0.93 (-10.83, 12.68)	0.46 (-3.81, 4.72)
**Daily Hospital Census**			
0-99	Ref	Ref	Ref
100-199	-1.61 (-15.41, 12.20)	-4.65 (-16.85, 7.55)	4.07 (-1.61, 9.76)
≥200	-3.78 (-25.50, 17.95)	-3.33 (-23.34, 16.69)	0.88 (-6.93, 8.70)

CI=Confidence Interval; CAD=Coronary Artery Disease; MI=Myocardial Infarction; TIA=Transient Ischemic Attack; NIH=National Institute of Health; EMS=Emergency Medical System

a. The intercept represents the mean interval time for each model with all patient and hospital characteristics set as the reference category. Analysis outputs from these models are reported as minutes greater/less than the intercept.
b. Other includes American Indian/Alaska Native, Asian, Native Hawaiian or Pacific Islander, or Unable to Determine. Race and ethnicity were extracted from the medical record for inclusion in the registry.
c. National Institutes of Health Stroke Scale (NIHSS) results range from 0-42, with higher scores indicating greater stroke severity.

© 2024 Royan R et al. *JAMA Network Open*.

eTable 5. GEE Regression Results for the Door-to–Vessel or Perfusion Imaging (DTVP) and Vessel or Perfusion Imaging–to-Door (VPTD) Intervals

	Model 1 (without DTVP or VPTD included) Minutes (95% CI)	Model 2 (using DTVP as a covariate) Minutes (95% CI)	Model 3 (using VPTD as a covariate) Minutes (95% CI)
Intercept[a]	238.07 (222.56, 253.58)	185.83 (172.74, 198.92)	175.44 (169.08, 181.80)
**Door to Vessel or Perfusion Imaging (DTVP)**	—	1.16 (1.09, 1.22)	—
**Vessel or Perfusion Imaging to Door (VPTD)**	—	—	1.03 (1.02, 1.05)
**DEMOGRAPHICS**			
**Age (Years)**			
18-59	Ref	Ref	Ref
60-69	-7.58 (-13.72, -1.45)	-3.78 (-8.89, 1.32)	-3.06 (-5.78, -0.34)
70-79	-2.12 (-9.60, 5.36)	1.66 (-4.76, 8.07)	-3.20 (-5.99, -0.42)
80-110	4.97 (-1.78, 11.72)	3.66 (-1.87, 9.19)	0.99 (-1.94, 3.92)
**Sex**			
Male	Ref	Ref	Ref
Female	8.80 (3.74, 13.86)	5.25 (0.67, 9.83)	2.83 (0.95, 4.70)
**Race/Ethnicity**			
Black or African American non-Hispanic	16.10 (8.50, 23.69)	5.93 (-0.96, 12.81)	8.67 (5.34, 12.00)
Hispanic	3.54 (-4.33, 11.41)	-0.52 (-7.25, 6.21)	4.00 (-0.35, 8.36)
Other non-Hispanic[b]	3.71 (-5.75, 13.17)	3.77 (-5.16, 12.70)	-0.21 (-3.51, 3.10)
White non-Hispanic	Ref	Ref	Ref
**Medical History / Prior Meds**			
Hypertension	0.79 (-4.70, 6.28)	1.17 (-3.78, 6.12)	-0.31 (-2.57, 1.95)
Dyslipidemia	-0.79 (-13.32, -2.51)	0.45 (-3.61, 4.51)	-1.23 (-3.07, 0.60)
Diabetes	3.57 (-1.24, 8.37)	0.90 (-3.20, 5.01)	2.34 (0.19, 4.49)
Prior stroke	8.90 (2.67, 15.14)	7.20 (1.40, 13.01)	1.19 (-1.29, 3.66)
CAD / Prior MI	0.51 (-4.96, 5.98)	0.03 (-5.18, 5.23)	0.60 (-1.81, 3.01)
Smoking	3.75 (-4.15, 11.66)	1.16 (-5.44, 7.77)	2.30 (-0.92, 5.52)
Atrial fibrillation	-7.91 (-13.32, -2.51)	-6.26 (-10.95, -1.56)	-1.26 (-3.40, 0.89)
Heart failure	-2.27 (-9.45, 4.92)	-2.59 (-9.03, 3.85)	0.76 (-5.07, 6.60)
Prior TIA	-3.50 (-11.18, 4.19)	-0.70 (-7.69, 6.28)	-2.47 (-5.30, 0.36)
Peripheral vascular disease	6.23 (-13.50, 25.96)	9.31 (-10.63, 29.25)	-3.04 (-7.67, 1.59)
Carotid artery stenosis	6.63 (-7.82, 21.08)	-0.69 (-13.72, 12.35)	6.85 (-0.63, 14.33)
Prosthetic heart valve	-14.46 (-25.11, -3.80)	-14.56 (-23.29, -5.83)	0.76 (-5.07, 6.60)
Prior antithrombotic medication	-5.55 (-10.60, -0.50)	-3.70 (-8.09, 0.69)	-1.35 (-0.68, 3.14)
**ARRIVAL & CLINICAL DATA**			
**NIH Stroke Scale Score[c]**			
0-6	Ref	Ref	Ref
7-12	-36.68 (-43.41, -29.95)	-20.42 (-26.33, -14.51)	-13.33 (-16.16, -10.50)
13-19	-53.32 (-60.37, -46.26)	-31.33 (-37.47, -25.20)	-17.76 (-20.75, -14.78)
≥20	-52.01 (-59.29, -44.74)	-33.18 (-39.30, -27.05)	-14.91 (-17.97, -11.86)
**Arrival Mode / Time**			
Private arrival mode	Ref	Ref	Ref
EMS no pre-notification	0.35 (-7.89, 8.59)	-2.73 (-9.90, 4.44)	2.72 (-1.22, 6.67)

© 2024 Royan R et al. *JAMA Network Open*.

EMS pre-notification	-18.46 (-25.75, -11.17)	-6.72 (-13.22, -0.22)	-9.79 (-12.45, -7.12)
**After Hours**	3.24 (-1.27, 7.75)	1.74 (-2.22, 5.69)	1.23 (-0.68, 3.14)
**During Pandemic**	1.01 (-4.48, 6.50)	8.21 (3.41, 13.00)	-6.42 (-8.72, -4.12)
**TRANSFERRING HOSPITAL CHARACTERISTICS**			
**Primary Stroke Center**	-8.65 (-16.32, -0.98)	-8.15 (-14.39, -1.92)	-0.43 (-3.90, 3.04)
**Location**			
Rural	Ref	Ref	Ref
Urban	-15.89 (-25.17, -6.60)	-9.52 (-17.15, -1.90)	-4.84 (-9.29, -0.39)
**Annual Thrombolysis Volume**			
0-9	Ref	Ref	Ref
10-19	-6.61 (-16.63, 3.40)	-6.46 (-14.68, 1.76)	-0.42 (-5.19, 4.35)
20-29	-9.31 (-21.56, 2.93)	-6.92 (-16.90, 3.05)	-2.02 (-7.74, 3.70)
30-126	-9.41 (-23.05, 4.22)	-2.37 (-13.51, 8.78)	-6.66 (-12.70, -10.50)
**Teaching Status**			
Non-Teaching	Ref	Ref	Ref
Teaching	4.05 (-4.08, 12.18)	1.09 (-5.67, 7.84)	2.71 (-1.21, 6.63)
**Daily Hospital Census**			
0-99	Ref	Ref	Ref
100-199	4.05 (-5.14, 13.25)	2.81 (-4.26, 9.87)	0.67 (-3.75, 5.09)
≥200	-6.27 (-17.94, 5.40)	-6.39 (-15.50, 2.72)	-0.47 (-5.94, 5.00)

CI=Confidence Interval; CAD=Coronary Artery Disease; MI=Myocardial Infarction; TIA=Transient Ischemic Attack; NIH=National Institute of Health; EMS=Emergency Medical System

a. The intercept represents the mean interval time for each model with all patient and hospital characteristics set as the reference category. Analysis outputs from these models are reported as minutes greater/less than the intercept.
b. Other includes American Indian/Alaska Native, Asian, Native Hawaiian or Pacific Islander, or Unable to Determine. Race and ethnicity were extracted from the medical record for inclusion in the registry.
c. National Institutes of Health Stroke Scale (NIHSS) results range from 0-42, with higher scores indicating greater stroke severity.

© 2024 Royan R et al. *JAMA Network Open*.

eTable 6. GEE Regression Results for the Door-to-Thrombolysis (Needle) (DTN) and Thrombolysis (Needle)-to-Door (NTD) Intervals

	**Model 1 (without DTN or NTD included) Minutes (95% CI)**	**Model 2 (using DTN as a covariate) Minutes (95% CI)**	**Model 3 (using NTD as a covariate) Minutes (95% CI)**
Intercept[a]	182.90 (171.18, 194.61)	158.76 (147.49, 170.02)	154.59 (150.41, 158.77)
**Door to Needle (DTN)**	—	0.96 (0.91, 0.99)	—
**Needle to Door (NTD)**	—	—	0.99 (0.99, 1.00)
**DEMOGRAPHICS**			
**Age (Years)**			
18-59	Ref	Ref	Ref
60-69	-6.88 (-12.56, -1.20)	-5.03 (-10.40, 0.34)	-1.99 (-4.12, 0.14)
70-79	-2.33 (-8.73, 4.08)	1.38 (-4.91, 7.66)	-3.86 (-5.56, -2.15)
80-110	1.49 (-5.03, 8.02)	2.58 (-3.67, 8.83)	-1.10 (-2.86, 0.67)
**Sex**			
Male	Ref	Ref	Ref
Female	7.09 (2.57, 11.61)	3.60 (0.64, 7.85)	3.70 (2.24, 5.16)
**Race/Ethnicity**			
Black or African American non-Hispanic	9.52 (1.29, 17.75)	5.57 (-2.34, 13.47)	4.01 (2.15, 5.87)
Hispanic	2.83 (-4.91, 10.57)	-0.57 (-8.52, 7.39)	3.20 (0.30, 6.10)
Other non-Hispanic[b]	8.31 (-3.61, 20.24)	6.70 (-4.91, 18.31)	1.66 (-0.73, 4.04)
White non-Hispanic	Ref	Ref	Ref
**Medical History / Prior Meds**			
Hypertension	0.11 (-5.04, 5.25)	-0.84 (-5.81, 4.13)	0.93 (-0.38, 2.25)
Dyslipidemia	1.36 (-3.42, 6.14)	1.68 (-2.86, 6.22)	-0.36 (-1.85, 1.14)
Diabetes	3.58 (-1.00, 8.16)	2.78 (-1.51, 7.06)	0.85 (-0.84, 2.56)
Prior stroke	8.25 (1.91, 14.59)	6.64 (0.51, 12.77)	1.69 (0.09, 3.28)
CAD / Prior MI	-0.12 (-5.97, 5.73)	-1.00 (-6.68, 4.68)	0.94 (-0.80, 2.67)
Smoking	-2.92 (-10.43, 4.58)	-2.16 (-9.14, 5.10)	-0.73 (-2.40, 0.95)
Atrial fibrillation	0.68 (-5.40, 6.76)	-1.94 (-7.88, 3.99)	2.70 (1.06, 4.33)
Heart failure	-0.17 (-6.75, 6.42)	-1.26 (-7.62, 5.10)	1.19 (-0.69, 3.08)
Prior TIA	5.83 (-4.62, 16.27)	4.75 (-5.40, 14.90)	1.16 (-1.04, 3.35)
Peripheral vascular disease	-1.87 (-11.76, 8.01)	-4.64 (-14.10, 4.81)	2.94 (-0.96, 6.83)
Carotid artery stenosis	-1.77 (-13.64, 10.10)	-4.05 (-15.96, 7.87)	2.40 (-0.86, 5.65)
Prosthetic heart valve	-7.56 (-20.43, 5.30)	-12.92 (-25.10, -0.74)	5.82 (0.36, 11.28)
Prior antithrombotic medication	-4.43 (-9.62, 0.77)	-2.87 (-7.70, 1.96)	-1.60 (-3.44, 0.24)
**ARRIVAL & CLINICAL DATA**			
**NIH Stroke Scale Score[c]**			
0-6	Ref	Ref	Ref
7-12	-16.53 (-22.66, -10.40)	-7.01 (-12.89, -1.12)	-9.92 (-11.61, -8.23)
13-19	-35.06 (-41.83, -28.29)	-22.14 (-28.77, -15.50)	-13.70 (-15.41, -11.98)
≥20	-36.90 (-43.51, -30.29)	-25.77 (-32.16, -19.39)	-11.85 (-13.62 (-10.07)
**Arrival Mode / Time**			
Private arrival mode	Ref	Ref	Ref
EMS no pre-notification	3.93 (-3.55, 11.42)	3.26 (-3.95, 10.46)	0.62 (-1.62, 2.87)
EMS pre-notification	-9.39 (-15.79, -3.00)	-2.12 (-8.13, 3.89)	-7.62 (-9.68, -5.57)

© 2024 Royan R et al. *JAMA Network Open*.

**After Hours**	5.20 (1.13, 9.27)	1.05 (-2.93, 5.02)	4.37 (3.07, 5.67)
**During Pandemic**	11.16 (6.30, 16.03)	12.15 (7.70, 16.60)	-0.96 (-2.73, 0.81)
**TRANSFERRING HOSPITAL CHARACTERISTICS**			
**Primary Stroke Center**	-3.12 (-10.28, 4.05)	-1.15 (-8.02, 5.72)	-1.66 (-3.69, 0.37)
**Location**			
Rural	Ref	Ref	Ref
Urban	5.47 (-2.99, 13.94)	8.62 (0.45, 16.80)	-3.30 (-5.62, -0.98)
**Annual Thrombolysis Volume**			
0-9	Ref	Ref	Ref
10-19	-6.70 (-15.44, 2.03)	-1.08 (-9.42, 7.27)	-5.88 (-8.54, -3.22)
20-29	-10.74 (-22.19, 0.70)	-0.56 (-11.56, 10.44)	-10.83 (-13.60, -8.07)
30-126	-12.23 (-24.89, 0.44)	-1.56 (-13.73, 10.61)	-11.13 (-14.59, -7.68)
**Teaching Status**			
Non-Teaching	Ref	Ref	Ref
Teaching	1.62 (-5.79, 9.02)	2.30 (-4.82, 9.41)	-1.05 (-3.19, 1.10)
**Daily Hospital Census**			
0-99	Ref	Ref	Ref
100-199	0.11 (-8.27, 8.49)	-1.11 (-9.25, 7.02)	1.21 (-1.16, 3.57)
≥200	-8.74 (-19.12, 1.64)	-7.71 (-17.62, 2.21)	-1.15 (-4.17, 1.87)

CI=Confidence Interval; CAD=Coronary Artery Disease; MI=Myocardial Infarction; TIA=Transient Ischemic Attack; NIH=National Institute of Health; EMS=Emergency Medical System

a. The intercept represents the mean interval time for each model with all patient and hospital characteristics set as the reference category. Analysis outputs from these models are reported as minutes greater/less than the intercept.
b. Other includes American Indian/Alaska Native, Asian, Native Hawaiian or Pacific Islander, or Unable to Determine. Race and ethnicity were extracted from the medical record for inclusion in the registry.
c. National Institutes of Health Stroke Scale (NIHSS) results range from 0-42, with higher scores indicating greater stroke severity.

© 2024 Royan R et al. *JAMA Network Open*.
