# Supplementary material for: Emergency Department Process Times and Door-In–Door-Out Times in Interhospital Transfers After Acute Ischemic Stroke
Source: JAMA Netw Open. 2024 Sep 3;7(9):e2431183. doi: 10.1001/jamanetworkopen.2024.31183 (PMC11372507; doi:10.1001/jamanetworkopen.2024.31183)
Supplement: Supplement 2. — Data Sharing Statement [file jamanetwopen-e2431183-s002.pdf]

Data Sharing Statement

Royan. Emergency Department Process Times and Door-In–Door-Out Times in Interhospital Transfers After Acute Ischemic Stroke. *JAMA Netw Open*. Published September 03, 2024. doi:10.1001/jamanetworkopen.2024.31183

Data
Data available: No

Additional Information
Explanation for why data not available: Data is available upon request from the American Heart Association.
